# Genome wide expression analysis of CBS domain containing proteins in *Arabidopsis thaliana *(L.) Heynh and *Oryza sativa *L. reveals their developmental and stress regulation

**DOI:** 10.1186/1471-2164-10-200

**Published:** 2009-04-28

**Authors:** Hemant R Kushwaha, Anil K Singh, Sudhir K Sopory, Sneh L Singla-Pareek, Ashwani Pareek

**Affiliations:** 1 Centre for Computational Biology and Bioinformatics, School of Information Technology, Jawaharlal Nehru University, New Delhi 110067, India; 2 Plant Molecular Biology, International Centre for Genetic Engineering and Biotechnology, New Delhi 110067, India; 3 Stress Physiology and Molecular Biology, School of Life Sciences, Jawaharlal Nehru University, New Delhi 110067, India

## Abstract

**Background:**

In *Arabidopsis thaliana *(L.) Heynh and *Oryza sativa *L., a large number of genes encode proteins of unknown functions, whose characterization still remains one of the major challenges. With an aim to characterize these unknown proteins having defined features (PDFs) in plants, we have chosen to work on proteins having a cystathionine β-synthase (CBS) domain. CBS domain as such has no defined function(s) but plays a regulatory role for many enzymes and thus helps in maintaining the intracellular redox balance. Its function as sensor of cellular energy has also been widely suggested.

**Results:**

Our analysis has identified 34 CBS domain containing proteins (CDCPs) in *Arabidopsis *and 59 in *Oryza*. In most of these proteins, CBS domain coexists with other functional domain(s), which may indicate towards their probable functions. In order to investigate the role(s) of these CDCPs, we have carried out their detailed analysis in whole genomes of *Arabidopsis *and *Oryza*, including their classification, nomenclature, sequence analysis, domain analysis, chromosomal locations, phylogenetic relationships and their expression patterns using public databases (MPSS database and microarray data). We have found that the transcript levels of some members of this family are altered in response to various stresses such as salinity, drought, cold, high temperature, UV, wounding and genotoxic stress, in both root and shoot tissues. This data would be helpful in exploring the so far obscure functions of CBS domain and CBS domain-containing proteins in plant stress responses.

**Conclusion:**

We have identified, classified and suggested the nomenclature of CDCPs in *Arabidopsis *and *Oryza*. A comprehensive analysis of expression patterns for CDCPs using the already existing transcriptome profiles and MPSS database reveals that a few CDCPs may have an important role in stress response/tolerance and development in plants, which needs to be validated further through functional genomics.

## Background

All eukaryotic genomes sequenced so far, contain a number of genes that encode for proteins whose functions are still unknown. These proteins have been documented to be induced under specific set of conditions and participate in protein-protein interactions and/or sometimes are also associated with mutant phenotypes [1]. These proteins with unknown functions are either called **p**roteins with **o**bscure **f**eatures (POFs) when they contain no previously defined domains/motifs, or **p**roteins with **d**efined **f**eatures (PDFs) when they contain at least one previously defined domain/motif [1,2]. A protein domain is an evolutionarily conserved unit of protein sequence that can evolve, function and exist independently of the rest of the protein chain. In general, each domain is assumed to perform a specific function. An identical domain may appear in evolutionarily and functionally unrelated proteins, and therefore it is challenging to relate the presence of a domain with overall functionality of the protein. One of the possible approaches to address this important issue is to use the microarray data as a tool to predict the function of proteins having unknown functions, as suggested [3]. Recently, a number of these kinds of proteins have been characterized in *Arabidopsis *and *Oryza *using transcriptome studies as well as functional genomics tools, by raising transgenic plants. It has been reported that some of these proteins of unknown function(s) can indeed improve tolerance of transgenic plants to oxidative stress [4].

To understand the probable mechanism of abiotic stress tolerance in *Oryza sativa*, we have made an attempt to characterize several unknown members of the stress responsive machinery [5]. A group of these proteins of unknown functions were found to have cystathionine-β-synthase (CBS) domain and were differentially regulated in the contrasting genotypes of rice indicating towards their probable role in salinity tolerance. Thus, we assume that these proteins may be participating in known pathways and networks and/or be involved in basic or specialized processes and also might comprise new and undiscovered pathways.

**Figure 1:**
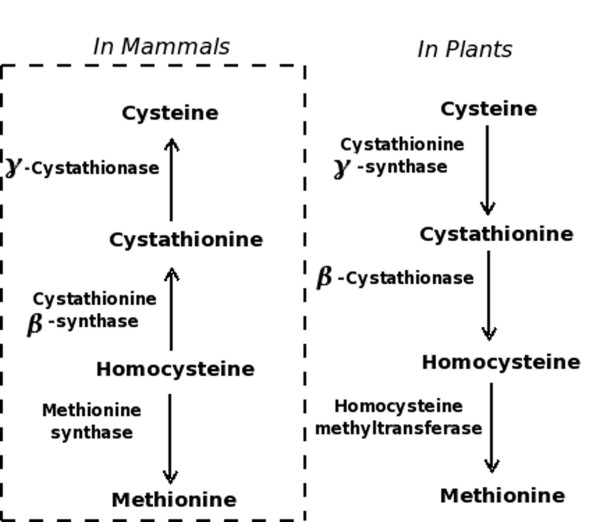
**Comparison of transulfuration pathway in plants and mammals showing role of Cystathionine β-synthase enzyme**.

CBS domains are found to be associated with several proteins of unrelated functions, such as **i**nosine-5'-**m**onophosphate **d**e**h**ydrogenase (IMPDH), **AMP**-activated **p**rotein **k**inase (AMPK), **c**h**l**oride **c**hannels (CLC) and **c**ystathionine-β-**s**ynthase (CBS). The importance of CBS domain was realized by the observation that point mutations in the CBS domain cause several hereditary diseases in humans [6]. CBS domain was first discovered by Bateman [7] in the genome of the archaebacterium *Methanococcus jannaschii *as a conserved domain in a group of proteins. CBS domain exists not only in archaebacterial proteins, but also in eubacterial and eukaryotic proteins [6]. The name of the CBS domain was coined after its discovery in human CBS enzyme, which is the first enzyme involved in the reverse transsulfuration pathway in which homocysteine is converted to cysteine via cystathionine. In plants and bacteria, transsulfuration pathway operates in forward direction leading to conversion of cysteine to homocysteine by the action of cystathionine-γ-synthase and β-cystathionase. While in mammals, reverse transsulfuration pathway is found in which cysteine is derived from homocysteine by CBS and γ-cystathionase enzymes (Figure 1). Yeast and some archaebacteria possess both transsulfuration pathways [8].

In CBS protein, C-terminal CBS domain exerts an autoinhibitory effect on the CBS activity, while binding of SAM (S-adenosylmethionine) with CBS domain induces a conformational change which relieves the autoinhibitory effect. Mutation in CBS domains abolish or strongly reduce activation by SAM and cause homocystinuria. CBS domain of γ-subunit of AMPK acts as sensor of cellular energy status and mutations cause a glycogen storage disease, which is clinically expressed as a familial hypertrophic cardiomyopathy (Wolff-Parkinson-White syndrome) [9-12]. Scott *et al*. [13] reported that CBS domain of IMPDH binds to ATP in a positive cooperative way and activates IMPDH. ATP binding and activation was abolished by a point mutation which corresponds to the mutation causing retinitis pigmentosa [14]. Earlier research shows that the CBS module in ATP-binding cassette transporter OpuA constitutes the ionic strength sensor whose activity is modulated by the C-terminal anionic tail [15]. In CLCs, function of CBS domain remains unresolved and controversial. However, it has been shown that CBS domains in human CLCs are required for its function and/or expression because mutations in the CBS domain of CLCs cause diseases due to CLC dysfunction [16-21]. However, in plants information available on CLCs is very limited. The first CLC gene (*CLC-Nt1*) was identified from tobacco [22], thereafter CLC genes were identified and characterized from *Arabidopsis *[23-25] and rice [26], but function of CBS domain in these CLC channel genes has not been resolved.

The availability of complete genome sequence along with the microarray expression and Massively Parallel Signature Sequencing (MPSS) data makes *Arabidopsis *an ideal plant for study of newly identified protein family [27]. In the present work, we have performed genome wide analysis in the two highly finished plant genomes i.e. *Arabidopsis thaliana *and *Oryza sativa *where we have identified and classified the CDCPs based on their conserved features. To further establish their possible involvement in development and abiotic stresses, we have analyzed the expression of genes encoding CDCPs using MPSS database and already existing *Arabidopsis *microarray database .

## Results and Discussion

### Identification and classification of CBS domain containing proteins

In the present study, we provide information on the CDCPs in *Arabidopsis thaliana *and *Oryza sativa*. For this, the CDCPs were searched against the protein sequence database for *Arabidopsis *and *Oryza*, using the HMM profile of CBS domain obtained from Pfam database as described earlier [28]. We propose a systematic classification of all the CDCPs based on their structural features, as there are no pre-existing reports on classification of CDCPs, till date.

**Figure 2:**
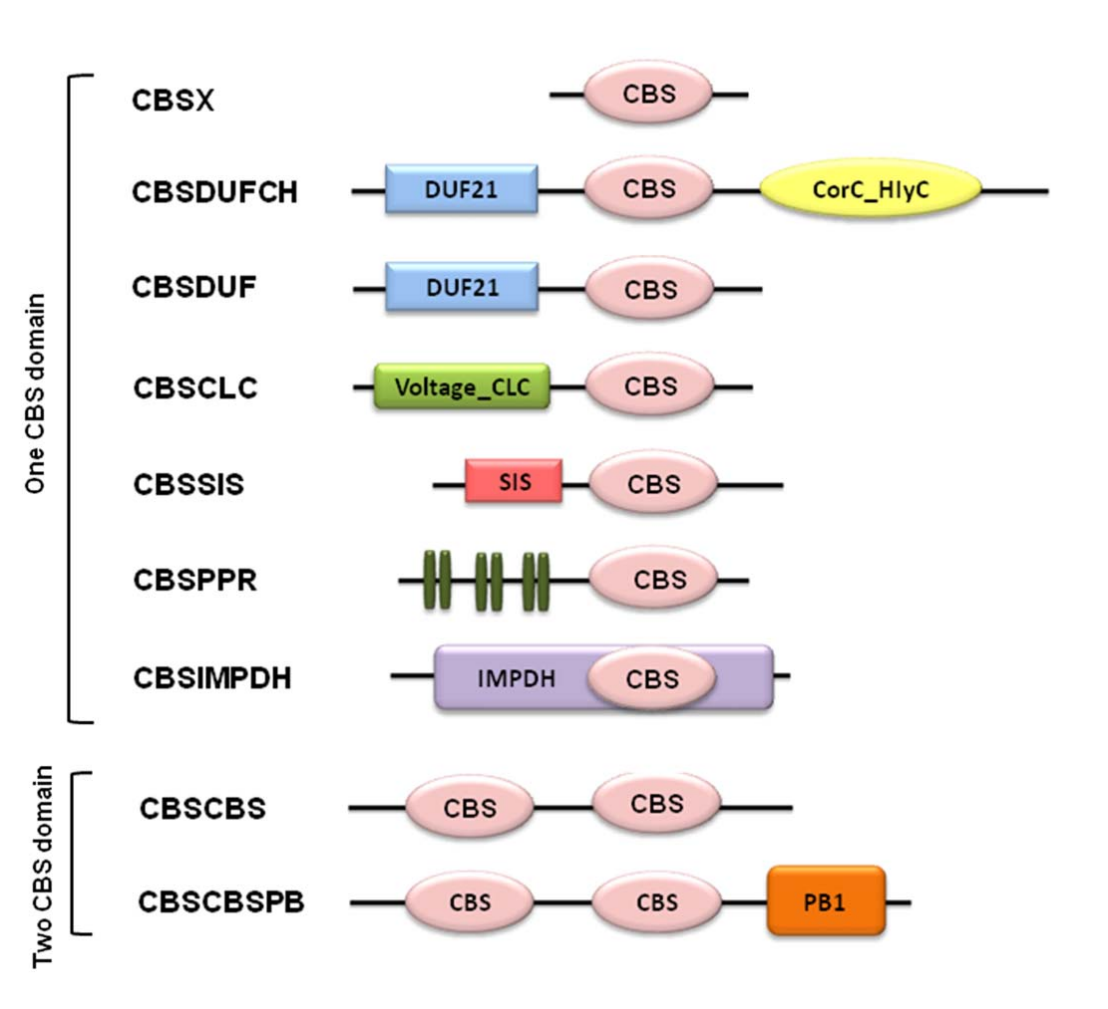
**Representation (unscaled) of the primary domain structure of the CDCP proteins in *Arabidopsis thaliana *and *Oryza sativa***. All the CDCPs were classified into two groups; single CBS domain containing proteins and two CBS domain containing proteins. Single CBS domain containing proteins were further classified into seven subgroups based on the additional domain(s) present in their sequences. Proteins containing only single CBS domain and no other functional domain were named as CBSX. Other proteins containing single CBS domain were named according to the presence of other functional domain. Two CBS domain containing proteins were classified into two subgroups. Proteins containing only two CBS domains and no other functional domain were named as CBSCBS and proteins containing two CBS domains and one PB1 domain were named as CBSCBSPB. The subfamily CBSIMPDH was observed only in *O. sativa*.

Whole genome analysis of *Arabidopsis *and *Oryza*, employing the standard bioinformatics tools (described in methods), identified a total of 34 proteins (encoded by 33 genes) in *Arabidopsis *and 59 proteins (encoded by 37 genes) in *Oryza *with a distinct CBS domain (Table 1). These proteins could be classified into two major groups comprising of proteins containing only a single and those with two CBS domains. Apart from CBS domain, some of these proteins also possess other structural domains based on which we have further classified these proteins into subgroups. Single CBS domain containing proteins were further classified into 6 subgroups in *Arabidopsis*, while in *Oryza *CDCPs were classified into 7 subgroups. Two CBS domain containing proteins were classified into 2 subgroups in both, *Arabidopsis *and *Oryza *(Figure 2). The other observed structural domains were: CorC_HlyC [29,30], voltage chloride channel (CLC) [31-33], **s**ugar **i**somerase (SIS) [34,35], **p**entatrico**p**eptide **r**epeats (PPR) [36-38], **P**hox and **B**em1p (PB1) [39-41] and **i**nosine **m**ono**p**hosphate **d**ehydrogenase (IMPDH) [14]. Some of the members of the CBS domains containing proteins also possess **d**omain of **u**nknown **f**unction (DUF21), while few other proteins only possess the CBS domain(s) in their sequences. In *Arabidopsis*, 25 proteins containing a single CBS domain were found which are encoded by 24 genes since one gene encoded for two alternate splice variants, whereas in *Oryza *46 proteins containing a single CBS domain were found to be encoded by 37 genes. Interestingly, in *Oryza *an additional class of CDCPs having IMPDH domain was observed, which was not found in *Arabidopsis*. The existence of more number of CDCPs in *Oryza *is due to alternative splice events which correspond to the earlier report where it has been shown that in *Oryza *36,650 alternate splicing events affected 8,772 genes, while in *Arabidopsis *16,252 alternate splicing events affected 5,313 genes [42]. In case of two CBS domain containing proteins, 9 genes encode for 9 proteins in *Arabidopsis*, as no case of alternative splicing was observed, whereas in *Oryza *8 genes encode for 13 proteins. The nomenclature has been assigned according to the domain(s) present in the given sequence such as CBSX for proteins containing only a single CBS domain, CBSDUFCH1 for protein containing one CBS domain along with DUF and **C**orC_**H**lyC domains, CBSCLC for proteins containing one CBS domain and a CLC domain, CBSDUF for proteins containing one CBS domain and a DUF domain, CBSSIS for proteins containing one CBS domain and a SIS domain and CBSPPR for proteins containing one CBS and a PPR domain CBSIMPDH for proteins containing one CBS domain along with an IMPDH domain. Whereas, for two CBS domain containing proteins, CBSCBS nomenclature was given to the proteins containing only two CBS domains, and CBSCBSPB for proteins containing two CBS domains and one PB1 domain (Figure 2). A prefix At in case of *A. thaliana *and Os in case of *O. sativa *proteins was assigned. For convenience, the alternative spliced forms were named as the gene name followed by postscript alphabets such as 'a', 'b' and so on.

**Table 1:** CBS domain containing proteins in *Oryza sativa *and *Arabidopsis thaliana*. In *O. sativa*, the genes encoding CBS domain proteins were named according to their classification prefixed by 'Os' while in *A. thailana *genes were prefixed by 'At'. The alternative spliced forms were post fixed with the alphabets like 'a', 'b' and so on.

**Gene**	**Proteins**	**TIGR id**	**Locus**	**Coordinate**	**AA**
***Oryza sativa *sub japonica one CBS domain containing proteins**					

*OsCBSX1*	OsCBSX1	12008.m06246	LOC_Os08g22149.1	13226547 – 13234580	235

*OsCBSX2*	OsCBSX2	12009.m03643	LOC_Os09g02710.1	1223593 – 1220510	227

*OsCBSX3*	OsCBSX3a	12002.m10750	LOC_Os02g57280.1	35086529 – 35087614	212
	
	OsCBSX3b	12002.m33940	LOC_Os02g57280.4	35086529 – 35087614	199
	
	OsCBSX3c	12002.m33942	LOC_Os02g57280.5	35086529 – 35087614	197
	
	OsCBSX3d	12002.m33941	LOC_Os02g57280.6	35086529 – 35087614	190
	
	OsCBSX3e	12002.m33943	LOC_Os02g57280.7	35086529 – 35087614	190

*OsCBSX4*	OsCBSX4a	12003.m10243	LOC_Os03g52690.1	30158802 – 30157593	230
	
	OsCBSX4b	12003.m34922	LOC_Os03g52690.2	30158802 – 30157394	205
	
	OsCBSX4c	12003.m34923	LOC_Os03g52690.3	30158802 – 30157394	205
	
	OsCBSX4d	12003.m34924	LOC_Os03g52690.4	30158802 – 30157394	205
	
	OsCBSX4e	12003.m101489	LOC_Os03g52690.5	30158802 – 30157593	170
	
	OsCBSX4f	13103.m05744	LOC_Os03g52690.8	30203267 – 30199873	231

*OsCBSX5*	OsCBSX5	12004.m05803	LOC_Os04g05010.1	2416552 – 2414325	220

*OsCBSX6*	OsCBSX6	12001.m10692	LOC_Os01g44360.1	25775244 – 25777361	258

*OsCBSX7*	OsCBSX7a	12001.m10314	LOC_Os01g40420.2	23148281 – 23147344	228
	
	OsCBSX7b	12001.m97525	LOC_Os01g40420.3	23148281 – 23147344	228

*OsCBSX8*	OsCBSX8	12003.m11263	LOC_Os03g63940.1	36063946 – 36070376	493

*OsCBSX9*	OsCBSX9	12002.m05989	LOC_Os02g06410.1	3204142 – 3206178	421

*OsCBSX10*	OsCBSX10	12001.m10682	LOC_Os01g44250.1	25695370 – 25697808	407

*OsCBSX11*	OsCBSX11	12002.m09299	LOC_Os02g42640.1	25640848 – 25642222	417

*OsCBSX12*	OsCBSX12	12004.m10713	LOC_Os04g58310.1	34488888 – 34487614	398

*OsCBSCLC1*	OsCBSCLC1	12001.m12661	LOC_Os01g65500.2	38344981 – 38348540	784

*OsCBSCLC2*	OsCBSCLC2	12001.m11267	LOC_Os01g50860.1	29546209 – 29548214	424

*OsCBSCLC3*	OsCBSCLC3a	12002.m08607	LOC_Os02g35190.1	21150763 – 21145109	804
	
	OsCBSCLC3b	12002.m33720	LOC_Os02g35190.2	21150763 – 21145109	804

*OsCBSCLC4*	OsCBSCLC4	12003.m09895	LOC_Os03g48940.3	27815829 – 27811366	508

*OsCBSCLC5*	OsCBSCLC5	12004.m10413	LOC_Os04g55210.1	32601607 – 32606015	808

*OsCBSCLC6*	OsCBSCLC6a	12008.m06120	LOC_Os08g20570.1	12344418 – 12350373	796
	
	OsCBSCLC6b	13108.m09367	LOC_Os08g20570.2	12349765 – 12355720	797

*OsCBSCLC7*	OsCBSCLC7	12012.m06347	LOC_Os12g25200.1	14487588 – 14485027	625

*OsCBSCLC8*	OsCBSCLC8	12008.m07881	LOC_Os08g38980.1	24506315 – 24512370	750

*OsCBSCLC9*	OsCBSCLC9a	12002.m09922	LOC_Os02g48880.1	29898156 – 29893927	783
	
	OsCBSCLC9b	12002.m33841	LOC_Os02g48880.2	29898156 – 29894593	693

*OsCBSCLC10*	OsCBSCLC10a	13104.m11737	LOC_Os04g36560.1	21892144 – 21886597	800
	
	OsCBSCLC10b	13104.m11901	LOC_Os04g36560.2	21892144 – 21886597	800

*OsCBSDUFCH1*	OsCBSDUFCH1a	12003.m09051	LOC_Os03g39640.1	21984229 – 21972448	680
	
	OsCBSDUFCH1b	13103.m12995	LOC_Os03g39640.2	22028109 – 22016078	682

*OsCBSSIS1*	OsCBSSIS1	12002.m05984	LOC_Os02g06360.1	3179627 – 3182495	344

*OsCBSDUF1*	OsCBSDUF1a	12005.m07517	LOC_Os05g32850.1	19149758 – 19156919	528
	
	OsCBSDUF1b	12005.m27812	LOC_Os05g32850.2	19149758 – 19155394	507

*OsCBSDUF2*	OsCBSDUF2	12003.m09725	LOC_Os03g47120.1	26601005 – 26605975	420

*OsCBSDUF3*	OsCBSDUF3	12003.m05876	LOC_Os03g03430.1	1454844 – 1461230	518

*OsCBSPPR1*	OsCBSPPR1	12009.m05771	LOC_Os09g26190.1	15789767 – 15799283	587

*OsCBSIMPDH1*	OsCBSIMPDH1a	12003.m10596	LOC_Os03g56800.1	32304776 – 32309729	501
	
	OsCBSIMPDH1b	12003.m34939	LOC_Os03g56800.2	32304776 – 32308874	492

***Arabidopsis thaliana *one CBS domain containing proteins**					

*AtCBSX1*	AtCBSX1	68417.m05232	At4g36910.1	17390623–17393305	236

*AtCBSX2*	AtCBSX2	68417.m04840	At4g34120.1	16341094–16343651	238

*AtCBSX3*	AtCBSX3	68418.m01261	At5g10860.1	3428892–3430471	206

*AtCBSX4*	AtCBSX4	68414.m09375	At1g80090.1	30134997–30136833	402

*AtCBSX5*	AtCBSX5	68417.m03946	At4g27460.1	13732916–13734310	391

*AtCBSX6*	AtCBSX6	68414.m07407	At1g65320.1	24260925–24262136	425

*AtCBSDUFCH1*	AtCBSDUFCH1	68416.m01634	At3g13070.1	4191518–4195119	661

*AtCBSDUFCH2*	AtCBSDUFCH2	68414.m06415	At1g55930.1	20922385–20925891	653

*AtCBSCLC1*	AtCBSCLC1a	68414.m06366	At1g55620.1	20790872–20794855	585
	
	AtCBSCLC1b	68414.m06367	At1g55620.2	20790872–20794855	781

*AtCBSCLC2*	AtCBSCLC2	68418.m03129	At5g26240.1	9189527–9194734	792

*AtCBSCLC3*	AtCBSCLC3	68417.m05035	At4g35440.1	16835991–16839380	710

*AtCBSCLC4*	AtCBSCLC4	68418.m06178	At5g49890.1	20305346–20309576	779

*AtCBSCLC5*	AtCBSCLC5	68418.m04965	At5g40890.1	16398542–16402547	775

*AtCBSCLC6*	AtCBSCLC6	68418.m03944	At5g33280.1	12566510–12569737	763

*AtCBSCLC7*	AtCBSCLC7	68416.m03398	At3g27170.1	10025254–10028435	780

*AtCBSDUF1*	AtCBSDUF1	68417.m02197	At4g14240.1	8204342–8207340	494

*AtCBSDUF2*	AtCBSDUF2	68417.m02196	At4g14230.1	8200707–8203215	495

*AtCBSDUF3*	AtCBSDUF3	68415.m01625	At2g14520.1	6189275–6191730	423

*AtCBSDUF4*	AtCBSDUF4	68414.m00305	At1g03270.1	799191–802436	499

*AtCBSDUF5*	AtCBSDUF5	68418.m06551	At5g52790.1	21408943–21411585	500

*AtCBSDUF6*	AtCBSDUF6	68417.m04786	At4g33700.1	16176403–16179482	424

*AtCBSDUF7*	AtCBSDUF7	68414.m05240	At1g47330.1	17353490–17356733	527

*AtCBSSIS1*	AtCBSSIS1	68416.m06051	At3g54690.1	20257517–20259002	350

*AtCBSPPR1*	AtCBSPPR1	68418.m01237	At5g10690.1	3374394–3377431	580

***Oryza sativa *sub japonica two CBS domain containing proteins**					

*OsCBSX7*	OsCBSCBS1	12001.m97524	LOC_Os01g40420.1	23148281 – 23147344	450

*OsCBSCBS2*	OsCBSCBS2	12001.m13024	LOC_Os01g69240.1	40567292 – 40565692	435

*OsCBSCBS3*	OsCBSCBS3	12004.m08236	LOC_Os04g31340.1	18564463 – 18559754	425

*OsCBSCBS4*	OsCBSCBS4a	12004.m35295	LOC_Os04g32880.1	19671270 – 19664088	451
	
	OsCBSCBS4b	12004.m101520	LOC_Os04g32880.2	19671270 – 19664088	451
	
	OsCBSCBS4c	12004.m08385	LOC_Os04g32880.4	19668390 – 19664088	346
	
	OsCBSCBS4d	12004.m35296	LOC_Os04g32880.3	19671270 – 19665094	405
	
	OsCBSCBS4e	13104.m11890	LOC_Os04g32880.5	19686702 – 19679520	452

*OsCBSCBS5*	OsCBSCBS5	12001.m13010	LOC_Os01g69090.1	40484031 – 40480347	404

*OsCBSCBSPB1*	OsCBSCBSPB1	12001.m13039	LOC_Os01g69900.1	40712332 – 40715773	552

*OsCBSCBSPB2*	OsCBSCBSPB2	12011.m04890	LOC_Os11g06930.1	3419765 – 3414996	560

*OsCBSCBSPB3*	OsCBSCBSPB3	12001.m13334	LOC_Os01g73040.1	42704156 – 42708054	533

*OsCBSCBSPB4*	OsCBSCBSPB4	12012.m04708	LOC_Os12g07190.1	3535282 – 3530718	542

***Arabidopsis thaliana *two CBS domain containing proteins**					

*AtCBSCBSPB1*	AtCBSCBSPB1	68418.m07970	At5g63490.1	25435820–25439410	543

*AtCBSCBSPB2*	AtCBSCBSPB2	68415.m04480	At2g36500.1	15325107–15327231	536

*AtCBSCBSPB3*	AtCBSCBSPB3	68416.m05837	At3g52950.1	19645474–19647797	556

*AtCBSCBSPB4*	AtCBSCBSPB4	68418.m06258	At5g50530.1	20589102–20592159	548

*AtCBSCBSPB5*	AtCBSCBSPB5	68418.m06274	At5g50640.1	20622444–20625490	548

*AtCBSCBS1*	AtCBSCBS1	68416.m05299	At3g48530.1	17998417–18000748	424

*AtCBSCBS2*	AtCBSCBS2	68414.m08031	At1g69800.1	26277893–26279992	447

*AtCBSCBS3*	AtCBSCBS3	68414.m01006	At1g09020.1	2899918–2904818	487

*AtCBSCBS4*	AtCBSCBS4	68414.m01834	At1g15330.1	5274341–5275597	352

### Analysis of CBS domain containing proteins

Whole genome analysis of CDCPs in *Arabidopsis *reveals the presence of 24 genes which code for 25 proteins containing only single CBS domain, while in *Oryza *29 genes coding for 46 proteins containing only single CBS domain were found. In case of two CBS domain proteins, 9 genes code for 9 proteins in *Arabidopsis*, while in *Oryza *8 genes code for 13 proteins [Table 1]. These proteins were further classified on the basis of additional domain(s) present within the sequence. The different sub-classes of these proteins are as follows:

#### A. CBSX

A fraction of predicted CBS domain-containing proteins harbor only single CBS domain (PF00571). CDCPs have been reported to have the regulatory functions , however the biological significance of these domains remains to be elucidated. These CDCPs can act as binding domains for adenosine derivatives and may regulate the activity of attached enzymatic or other domains [43]. In some cases, these proteins may act as sensors of cellular energy status as they are activated by AMP and inhibited by ATP [44]. Recently, one of the *Arabidopsis *CBSX proteins (CDCP2) has been purified and crystallized [45]. In case of *Arabidopsis*, 6 genes were found to encode for 6 CBSX proteins, while in *Oryza*, a total of 22 CBSX proteins were categorized in this subgroup, which were encoded by 12 genes. In *Oryza*, all the *CBSX *genes code for only one protein, except for OsCBSX3, OsCBSX4 and OsCBSX7, which code for 5, 6 and 2 proteins, respectively through the alternate splicing mechanism.

#### B. CBSDUFCH

In case of *Arabidopsis*, this subgroup contains two proteins which are encoded by two genes, while in case of *Oryza*, two proteins are encoded by a single gene. These proteins contain a domain of unknown function (DUF21) (PF01595) at the N-terminus adjacent to a CBS domain and a CorC_HlyC domain (PF03471) at the C-terminus. DUF21 domain has no known function and is usually present at the N-terminus of the proteins adjacent to a CBS domain. The CorC_HlyC is a transport associated domain and is found at the C-terminus of the proteins. CorC_HlyC domain is also found in magnesium and cobalt efflux protein CorC and some of the Na^+^/H^+ ^antiporters. The function of this domain is unknown but it might be involved in modulating the transport of ion substrates .

#### C. CBSDUF

Proteins classified in this subgroup contain a DUF21 domain at the N-terminus, along with a CBS domain. In case of *Oryza*, this subgroup contains four proteins, which are encoded by three genes, as OsCBSDUF1 gene codes for two proteins whereas, in case of *Arabidopsis*, this subgroup contains seven proteins, which are encoded by seven genes.

### D. CBSCLC

These proteins belong to chloride channel protein (CLC) family which sustains a wide variety of cellular functions, including membrane excitability, synaptic communication, transepithelial transport, cell volume recognition, cell proliferation, and acidification of endosomes and lysosomes. Earlier chimeric and deletion approaches had suggested that CBS domain may influence gating of CLCs. Past studies have suggested that mutation in the CBS domain affects protein-protein interaction within CLC protein subunits as well as between two subunits of the dimer and that they influence the voltage dependence of gating through the common gate [46]. Role of CBS domain in the correct targeting (possibly related to correct folding) of the CLC is in accordance with previous studies with yeast CLC protein where mutation in the CBS domain abolished its localization to the late golgi that is seen upon its overexpression [47]. Earlier experiments have shown that certain mutations in CBS domain affect chloride channel gating but physiologically relevant regulatory role of CBS domains in CLC channels is yet to be established [46]. In an earlier report, 7 CLC genes were identified each from *Oryza and Arabidopsis *[48]. However, in the present study we have identified total 10 genes encoding 14 CLC proteins in *Oryza*. Whereas in case of *Arabidopsis*, our results are in accordance with the previous report as we found 8 CLC proteins encoded by 7 genes.

#### E. CBSSIS

Proteins containing sugar isomerase (SIS) domain along with a CBS domain have been classified in this subgroup. We have identified one CBSSIS gene encoding only one protein in cases of both, *Arabidopsis *and *Oryza*. The SIS domain is widespread and found in all species including, prokaryotic, archaebacterial and eukaryotic proteins. In general SIS domain is found in proteins that have a common role in phosphosugar isomerization as SIS domain functions by binding to the phosphosugars. The SIS domains are also found in a family of bacterial transcriptional regulators [34] as well as in a family of *Escherichia coli *iron transporters [49].

#### F. CBSPPR

In both *Arabidopsis *and *Oryza*, this subgroup is composed of a single gene encoding for a single protein, containing a pentatricopeptide repeat (PPR) motif and a CBS domain. The PPR motif, first described by Small *et al*. [36] is a degenerate 35 amino acid sequence closely related to the 34 amino acid tetratricopeptide repeats (TPR) motif. PPR repeat motifs are structural motifs encoded by a large number of genes in plants and other organisms, although the PPR gene family is greatly expanded in plants. It was hypothesized that this could be due to novel functions served by PPR proteins in plants that are not required in other organisms, or that PPR proteins replace functions performed by other genes in other organisms. Also, restoration of male fertility is a plant specific function encoded by PPR genes [50]. A genome-wide analysis of *Arabidopsis *PPR family proteins has identified 441 members and their further analysis revealed that PPR proteins play constitutive, often essential roles in mitochondria and chloroplast, probably via binding to organeller transcripts [51]. Many plant PPR genes that have been functionally annotated so far, are involved in either male fertility restoration through modification or silencing of cytotoxic mitochondrial transcripts, or post transcriptional modulation of plastid gene expression or plant embryogenesis and other plant developmental processes [52]. Interestingly, among large number of PPR proteins found in plants, only one PPR protein, each in *Oryza *and *Arabidopsis*, contains a CBS domain in the same reading frame. Occurrence of CBS domain along with PPR repeat in a protein suggests that this protein might be involved in the various cellular processes by sensing the cell energy status [13]. Structurally characterizing the full length protein (PPR + CBS domain) may shed light on this regulatory mechanism in plants.

#### G. CBSIMPDH

Proteins containing inosine-5'-monophosphate dehydrogenase (IMPDH) domain (PF00478) along with CBS domain has been classified in this subgroup. In *Oryza*, only one CBSIMPDH gene has been identified, which code for two CBSIMPDH proteins while in *Arabidopsis*, no member of this subgroup has been identified. Interestingly, in *Oryza *CBSIMPDH proteins, CBS domain lies within the IMPDH domain. IMPDH domain has been identified in the sequence of IMPDH enzyme, which is a key enzyme in the *de novo *guanosine nucleotide biosynthesis. Scott et al. [13] have shown that the CBS domain of IMPDH binds to ATP *in vitro *and that the tetrameric IMPDH binds ATP in a positive, cooperative way. They have also observed that IMPDH was activated by ATP, which was never reported earlier. This observation strongly supported the verity that ATP binding to the CBS domain allosterically activates IMPDH and consequently XMP synthesis. If so, this mechanism would couple the GTP/dGTP biosynthesis to the cellular energy status i.e., high ATP levels [6].

#### H. CBSCBS

In this subgroup, proteins containing only two CBS domains have been classified. In case of *Oryza*, 8 CBSCBS proteins have been identified, which are encoded by 5 different genes. The OsCBSX7 gene, which has been classified in CBSX subgroup, also codes for a protein OsCBSCBS1 containing two CBS domains. The OsCBSCBS4 gene encodes for four proteins through an alternative splicing mechanism whereas, in case of *Arabidopsis*, AtCBSCBS subgroup contains four genes, which encode four proteins.

#### I. CBSCBSPB

These proteins contain Phox/Bemp1 (PB1) domain along with the CBS domain. In case of *Oryza*, this subgroup possess 4 genes, which encode for 4 proteins, while in *Arabidopsis *5 CBSCBSPB genes encode for 5 proteins. The PB1 domains are present in many eukaryotic cytoplasmic signaling proteins. They are dimerization/oligomerization domains present in adaptor and scaffold proteins and kinases that serve to organize platforms that ensure specificity and fidelity during cellular signaling. Recently, a number of studies have provided valuable information on the structural details that govern binding between the different PB1 modules and explain how they direct the formation of different macromolecular signaling complexes [53]. Proteins containing the PB1 domain are conserved in animals, fungi, amoebae and plants, which participate in various biological processes [54]. The function of PB1 domain containing proteins in plants has not been reported so far. Presence of PB1 domain, along with a pair of CBS domains, in a single protein suggests that these proteins might be involved in cellular signaling processes through interaction with other proteins and/or ligands (ATP, ADP or SAM). Characterization of these proteins at physiological, molecular and structural level might shed some light on their functionality.

### Chromosomal distribution of CBS domain containing proteins

**Figure 3:**
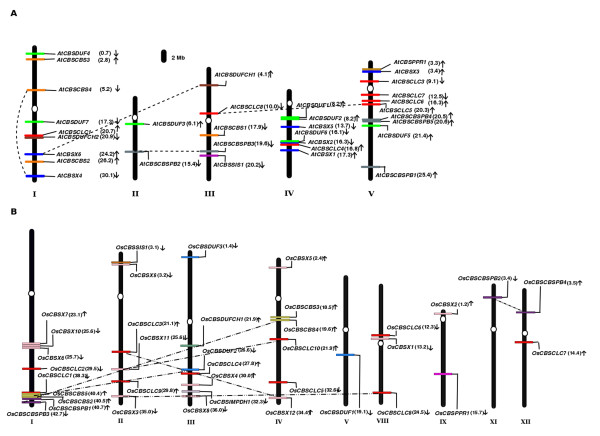
**Genomic distribution of CDCP genes on *Arabidopsis thaliana *(A) and *Oryza sativa *(B) chromosomes**. White ovals on the chromosomes (vertical bars) indicate the position of centromeres. Chromosome numbers are indicated at the bottom of each bar. The position of first exon of genes (in Mb) has been marked in the parentheses along with their names at the same location on chromosomes. Arrow marks the direction of the ORF specific to the gene encoding CDCP protein. In case of *Oryza*, only those chromosomes having CDCP genes are shown.

In *Arabidopsis*, the family of 33 CDCP genes was found to be distributed randomly on all the 5 chromosomes (Figure 3a), while in *Oryza *the family of 37 CDCP genes was distributed on 9 out of 12 chromosomes (Figure 3b). In case of *Arabidopsis*, maximum (10 in number) CDCP genes, were found to be located on chromosome V, followed by 9 on chromosome I, 7 on chromosome IV, 5 on chromosome III and 2 on chromosome II. While in *Oryza *maximum (9 in number) CDCP genes were found to be located on chromosome I, followed by 7 on chromosome III, 6 each on chromosome II and IV, 3 on chromosome VIII, 2 each on chromosomes IX and XII; and 1 each on chromosomes V and VII.

The distribution of the CDCP genes on the 5 chromosome of *Arabidopsis *and 9 chromosome of *Oryza*, at which they were found to be located, is not uniform. Their chromosomal distribution pattern reveals that some CDCP genes are found in clusters on certain chromosomes at various chromosomal regions. Occurrence of cluster of genes belonging to a family at certain chromosomes and chromosomal regions is common. Jain et al. [55] have reported that among the 19 auxin responsive (SAURs) genes present on chromosome IX, 17 are clustered together at a single locus in tandem. Similarly, genes encoding basic leucine zipper transcription factors (*OsbZIP*) have also been reported to be present in clusters at certain chromosomes and chromosomal regions [56]. In *Oryza*, 4 genes encoding two CBS domain containing proteins were found in close vicinity at chromosome I. Percent identity amongst all the four genes clustered was found to be in the range of 30 to 60%.

The sequence information and analysis of the *Arabidopsis *[57-60] and *Oryza *[61] whole genomes have revealed numerous large-scale segmental duplications. Several studies conclude that at least two rounds of duplications might have probably occurred in the *Arabidopsis *genome, with many losses and rearrangements, leaving a mosaic of "segmental duplications" or "duplication blocks" [61-65]. Analysis of CDCP genes has revealed that some of these genes have been duplicated during the process of evolution in both, *Arabidopsis *and *Oryza*. Figure 3a shows the duplicated CDCP genes in *Arabidopsis *and Figure 3b shows the duplicated CDCP genes in *Oryza*. In *Arabidopsis*, AtCBSX4 at chromosome I seem to be duplicated segmentally, and a domain duplication event might have occurred, which have resulted in the appearance of AtCBSCBS4 gene on the same chromosome. AtCBSDUFCH2 gene at chromosome I was observed to be duplicated to chromosome III as AtCBSDUFCH1. These duplicated genes show 85% identity at the nucleotide level and 79% identity at the protein level. AtCBSCLC8, present on the chromosome III seems to be duplicated segmentally to chromosome V as AtCBSCLC6 and at the same time the gene observed to have undergone inversion. AtCBSCBSPB2 present at chromosome II seems to be segmentally duplicated at chromosome III as AtCBSCBSPB3. These genes share 86% identity at the nucleotide level and 67% identity at the protein level. Interestingly in *Oryza*, it was observed that during the course of duplication the structural domains accumulate variations in order to adapt to new function. For example OsCBSDUF2 gene present on chromosome III got duplicated on chromosome I as OsCBSCBS5. In other instances the CDCP genes were duplicated but retained their structural domains, like OsCBSCBSPB2 present on chromosome XI got duplicated as OsCBSCBSPB4 on chromosome XII. OsCBSCLC9 on chromosome II was found to be duplicated as OsCBSCLC8 on chromosome VIII. OsCBSCBS2 located on chromosome I was observed to be duplicated as OsCBSCBS3 on chromosome IV.

### Sequence Analysis of CBS domain containing proteins

Sequence analysis of all the CDCPs shows an overall homology amongst their own respective groups (see Additional files: 1 and 2). Alignment of CBS domain sequences shows that the conserved domain also possesses some variations within themselves. Single CBS domain proteins of *Oryza *have been observed to have a percent identity of 55% to 60% within themselves, and 30% with that of *Arabidopsis *except for the AtCBSX1 which showed identity of more than 80% with OsCBSX1 and OsCBSX2. Similar identity pattern was also observed for the OsCBSCLC proteins, which showed identity ranging from 30% to 80% within their own subgroups and also with AtCBSCLC proteins. The OsCBSDUF proteins were observed to have identity ranging from 50% to 60% with the AtCBSDUF proteins. The OsCBSSIS1 protein shares 80% identity with AtCBSSIS1 and OsCBSPPR1 protein shares 60% identity with AtCBSPPR1 protein. OsCBSCBS proteins share 40% to 75% identity among themselves. The two CBS domain proteins also showed 50% to 70% identity with the other two CBS domain members. Apart from having variations, the CBS domain also accumulates large insertions. The large insertion or deletion might have helped the CBS domain proteins to evolve in order to perform specific functions. Results from the sequence alignment suggested that the sequences might have evolved according to the other functional domains present in the sequences which might have led to their specialized functions.

### Phylogenetic analysis of CBS domain containing proteins

**Figure 4:**
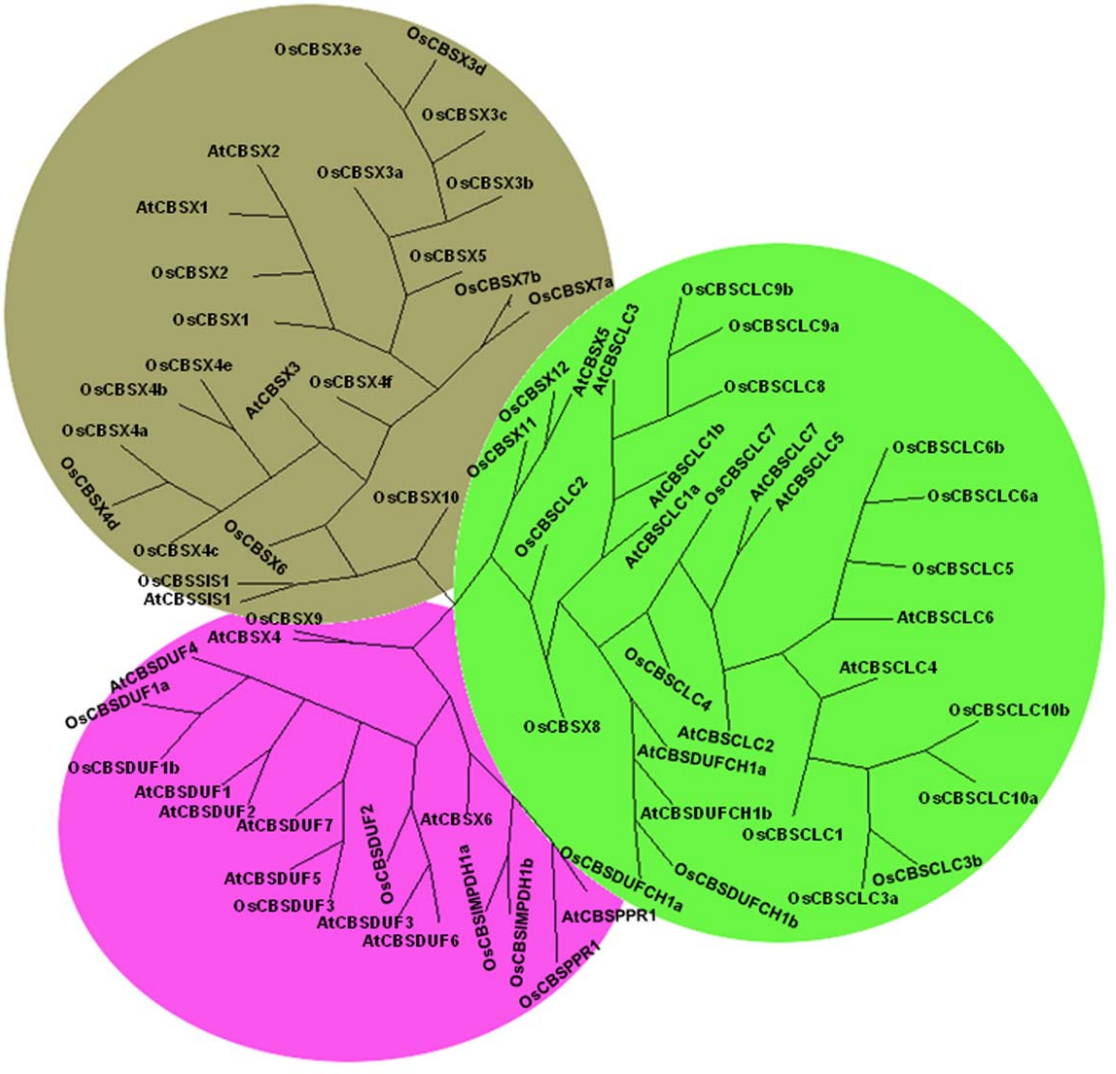
**The unrooted parsimonious tree of single CBS domain containing proteins in *A. thaliana *and *O. sativa *showing different clusters**. The tree was plotted using drawtree program of phylip software.

**Figure 5:**
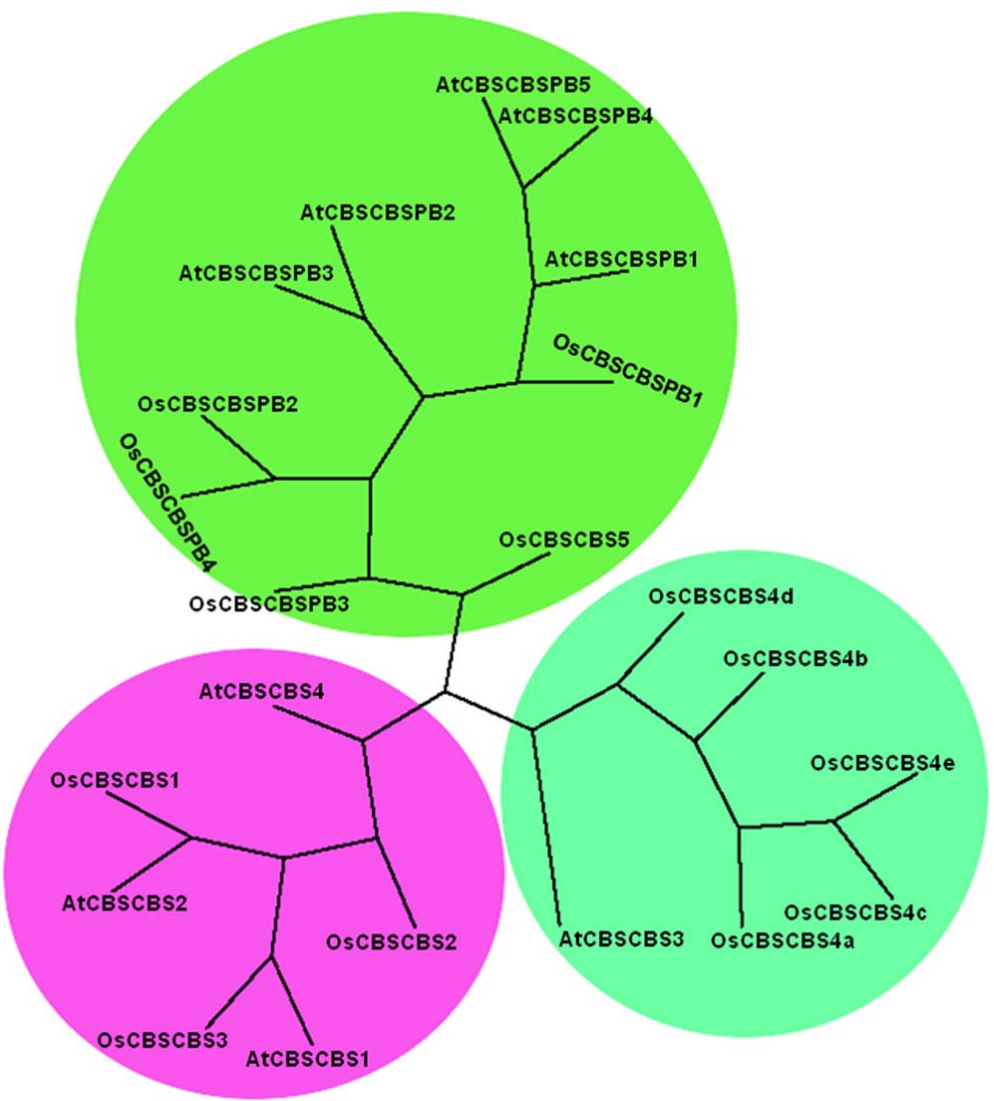
**The unrooted parsimonious tree of two CBS domain containing proteins in *A. thaliana *and *O. sativa *showing different clusters**. The tree was plotted using drawtree program of phylip software.

To study the phylogenetic relationship amongst the CDCPs in both *Arabidopsis *and *Oryza*, an unrooted tree was constructed from the alignment of full length protein sequences. Analysis of single CBS domain proteins in both *Arabidopsis *and *Oryza *revealed that all the single CBS domain containing proteins were divided into three clades (Figure 4). It was observed that all the OsCBSX and AtCBSX proteins clustered together in single clade, except for OsCBSX8, OsCBSX9, OsCBSX11, OsCBSX12, AtCBSX4, AtCBSX5 and AtCBSX6. The OsCBSX11 and OsCBSX12 proteins were found in the clade with CBSCLC proteins showing significant identity (52%) with that of the CBSCLC proteins. The OsCBSSIS1 and AtCBSSIS1 proteins share cluster with majority of OsCBSX and AtCBSX proteins. It was observed that OsCBSSIS1 and AtCBSSIS1 proteins share 40% identity with each other. All the OsCBSDUF and AtCBSDUF proteins were clustered together in the same clade. AtCBSPPR1, OsCBSPPR1 and OsCBSIMPDH proteins were also found to be lying in the same clade along with CBSDUF proteins. OsCBSIMPDH proteins share 30% to 44% identity with the CBSDUF proteins. Third clade comprises of the OsCBSCLC and AtCBSCLC proteins and it was also shared by OsCBSDUFCH1 and AtCBSDUFCH1 proteins. Analysis of two CBS domain containing proteins in *Arabidopsis *and *Oryza *clearly showed three clades (Figure 5). The OsCBSCBSPB and AtCBSCBSPB proteins were observed to be clustered together in one clade, while OsCBSCBS and AtCBSCBS proteins were found to be divided in two separate clades. The first clade represented all the CBSCBS proteins except for OsCBSCBS4 and AtCBSCBS3. The large number of alternative splicing observed in OsCBSCBS4 resulted in the separate clade showing the amount of variation adopted by these proteins with respect to the other OsCBSCBS proteins. When observed at the sequence level OsCBSCBS4 was found to have 50% to 60% identity with other CBSCBS members. OsCBSCBS5 protein was found to be closer to the OsCBSCBS4 protein showing an increase in the copy number of the protein in *Oryza *due to evolution which might be caused in order to adapt for specific function. These results suggest that the CBS domain containing proteins might have evolved differentially in order to adapt to the specific functions.

### MPSS analysis of genes encoding CBS domain containing proteins

**Figure 6:**
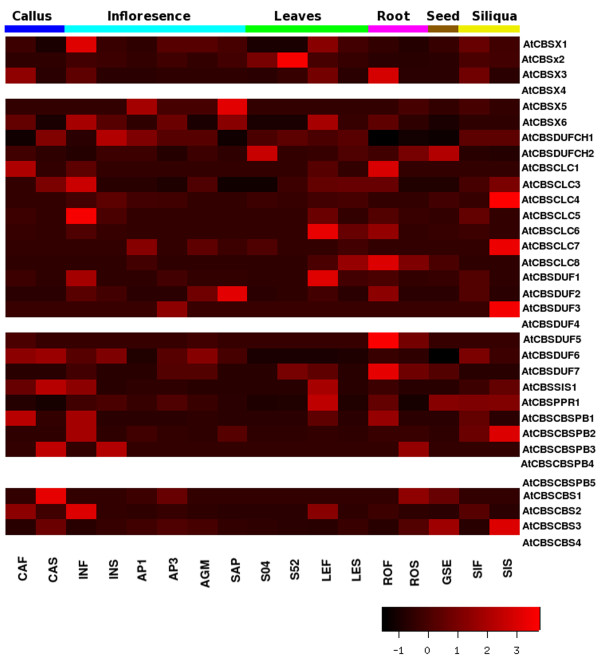
**Heatmap of the expression analysis from the MPSS data in different tissues of the *Arabidopsis thaliana***. The empty rows of heatmap correspond to the absence of transcript abundance values for the respective gene. The heatmap was made using gplots package of open source R software. The scale shows the Z-score, which is defined as "actual value" minus the mean of the group divided by the standard deviation.

**Figure 7:**
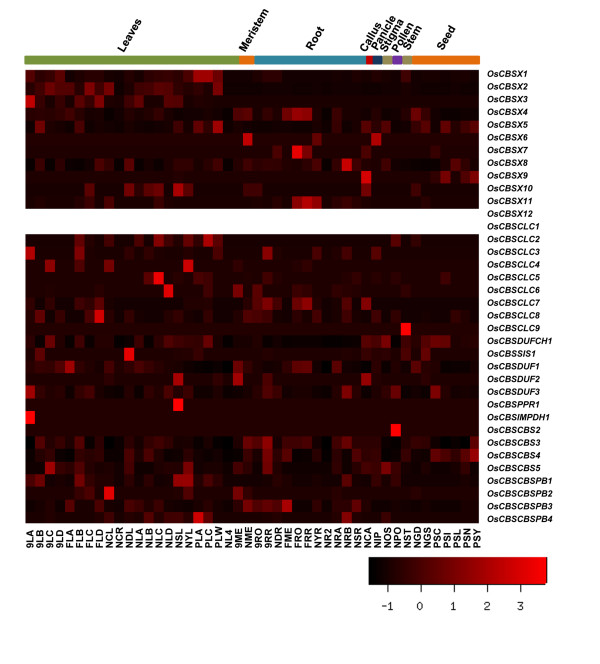
**Heatmap of the expression analysis from the MPSS data in different tissues of the *Oryza sativa***. The empty rows of heatmap correspond to the absence of transcript abundance values for the respective gene. The heatmap was made using gplots package of open source R software. The scale shows the Z-score, which is defined as "actual value" minus the mean of the group divided by the standard deviation.

Massively parallel signature sequencing (MPSS) provides a sensitive quantitative measure of gene expression for nearly all genes in the genome [66]. To study the expression of CDCP genes in various tissues/organs under different conditions, we extracted the information about the MPSS tags available for both 17 base and 20 base libraries representing 6 different parts of the plant from *Arabidopsis *MPSS Project  and *Oryza *MPSS project  (see Additional files: 3 and 4). The heatmaps generated from these data are presented as Figure 6 and 7.

Analysis of CDCP genes in *Arabidopsis and Oryza *showed that AtCBSX4 has no expression value in any of the tissues under any condition according to the MPSS database while in *Oryza *OsCBX12 has no expression values in the MPSS database. In *Arabidopsis*, genes encoding for proteins with CorC_HlyC functional domain, which has a major role in magnesium and cobalt efflux, along with CBS domain seems to be expressed more in siliqua, leaves and inflorescence. In *Oryza*, OsCBSDUFCH1 gene was observed to show more expression in leaves and other plant tissues, except roots. The gene AtCBSDUFCH1 is expressed more in inflorescence of ap1-10, ap3-6 mutants than in the normal condition of inflorescence. AtCBSDUFCH2, which is duplicated from AtCBSDUFCH1 on chromosome III, is expressed more in leaves and seed. In case of *Arabidopsis*, CBSCLC genes showed more expression in callus, roots and leaves overall in the MPSS analysis, while in case of *Oryza*, OsCBSCLC7 seems to be more expressed in root tissues, while other members were found to be expressed more in aerial part of the plant. It was observed that in TIGR ver 6, the data for OsCBSCLC10 was not available in the MPSS database. The CBSSIS1 gene seems to be expressed more in callus, normal inflorescence and leaves, while CBSPPR1 gene showed more expression in leaves, seed, siliqua and roots in *Arabidopsis*. In *Oryza *CBSSIS1, CBSPPR1 and CBSIMPDH1 genes were observed to maintain a constant level of expression in all the plant tissues.

Among the groups of genes having two CBS domains, MPSS analysis showed higher expression for all the CBSCBS genes in all the plant parts in *Oryza*, whereas in case of *Arabidopsis*, AtCBSCBS4 does not show expression in any of the plant part considered for the MPSS analysis. AtCBSCBS2 showed enhanced levels of expression in normal inflorescence, leaves and callus. While, AtCBSCBS3 showed expression in other inflorescence such as ap1-10, ap3-6 and agamous mutant conditions but does not show any expression in the normal inflorescence.

In *Arabidopsis*, AtCBSCBSPB1, AtCBSCBSPB2 and AtCBSCBSPB3 showed expression in MPSS analysis. Whereas in *Oryza*, OsCBSCBSPB3 was observed to show expression in the meristematic tissues and root while rest of the genes of this subclass were found to have been expressed in leaves also. In *Arabidopsis*, AtCBSCBSPB1 showed more expression in callus, root and normal inflorescence, while AtCBSCBSPB2 showed expression only in normal inflorescence and siliqua. This data indicates that CDCP genes exhibit a strict tissue specific expression which is also developmentally controlled.

### Expression profiles of genes encoding CBS domain containing proteins under various stresses

**Figure 8:**
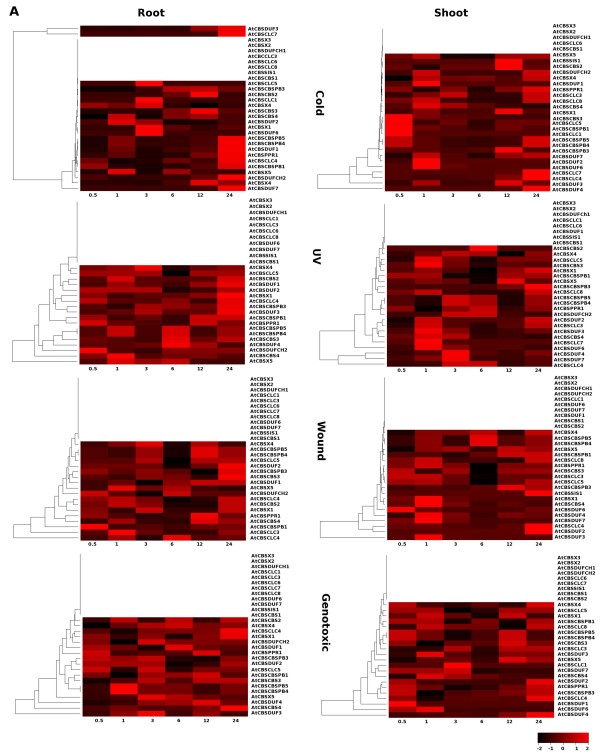
**Heatmap analysis of CDCP genes using microarray data obtained from TAIR 8**. The microarray data of the selected gene expression for various abiotic stress conditions such as cold, UV, wound, genotoxic stress were retrieved from TAIR (ver 8). The datasets obtained were corresponding to roots and shoots tissue at different time sets of stress namely 30 min, 1 h, 3 h, 6 h, 12 h and 24 h and analyzed with respect to the control. The empty rows in the heatmap show the unaltered behavior of the respective gene with respect to the control. The hierarchical clustering was performed and heatmaps were generated using gplots package of open source R software respectively. The scale shows the Z-score, which is defined as "actual value" minus the mean of the group divided by the standard deviation.

**Figure 9:**
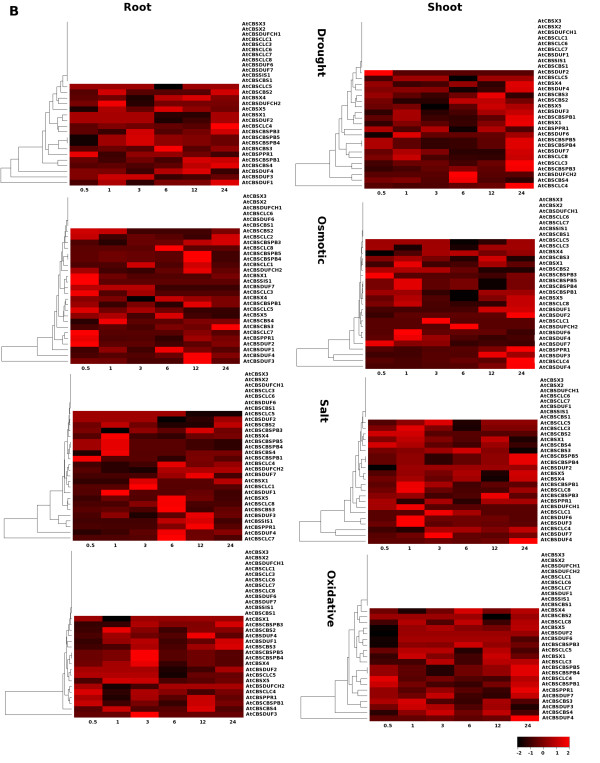
**Heatmap analysis of CDCP genes using microarray data obtained from TAIR 8**. The microarray data of the selected gene expression for various abiotic stress conditions such as drought, osmotic, salt and oxidative stress were retrieved from TAIR (ver 8). The datasets obtained were corresponding to roots and shoots tissue at different time sets of stress namely 30 min, 1 h, 3 h, 6 h, 12 h and 24 h and analyzed with respect to the control. The empty rows in the heatmap show the unaltered behavior of the respective gene with respect to the control. The hierarchical clustering was performed and heatmaps were generated using gplots package of open source R software respectively. The scale shows the Z-score, which is defined as "actual value" minus the mean of the group divided by the standard deviation.

To examine the expression of CBS domain containing proteins under various abiotic stress conditions in *Arabidopsis*, we took advantage of the available data on transcriptional profiling . Analysis of microarray data indicated that some of the CDCP genes are regulated by various abiotic stress conditions (Figure 8 and 9).

Thomashow [67] has shown that model plant *Arabidopsis *may express as many as hundreds of cold shock proteins under cold stress condition. CDCP genes showed altered expression at 24 hr cold stress in root with respect to the control, while in shoot some of the CDCP genes showed early upregulation within 30 min and 1 hr of cold stress. Some of the CDCP genes also showed up regulation at 12 hr to 24 hr cold stress conditions. AtCBSSIS1 gene was found to be upregulated in shoots under cold and wound stresses, while in roots, upregulation was observed only under osmotic and salt stresses. In roots, AtCBSDUF3 and AtCBSCLC7 showed upregulation in cold stress condition at 12 hour and 24 hour. Under cold stress condition AtCBSCBSPB4 and AtCBSCBSPB5 showed upregulation within 24 hrs in roots but in shoots they showed biphasic upregulation i.e. their expression was upregulated within 30 min of stress, followed by downregulation of expression and it was again upregulated within 24 hr of the stress exposure. High (intense) light stress causes the formation of oxygen radicals in chloroplasts and has the potential to damage them. However, plants are able to respond to this stress and protect chloroplasts by various means, including transcriptional regulation in the nucleus. Although the corresponding signaling pathway is unknown, there have been attempts to study its regulation [68]. When exposed to UV light, CDCP genes showed upregulation at 24 hr exposure in roots (also expressed at early timepoints), while in shoot, UV light exposure from 1 to 3 hrs is found to be sufficient to induce the expression of CDCP genes. Most of the CDCP genes maintain a constant level of expression during all the time period of the UV exposure suggesting that these genes might play crucial role(s) in the light sensing mechanism. The complex responses of plants to wounding have been extensively studied in recent years, and numerous wound-responsive genes have been identified in *Arabidopsis *[69]. Wounding stress in shoot showed comparable expression at all the time points while in roots CDCP genes showed expression at 1 hr and 24 hr of exposure to stress. When exposed to the genotoxic stress, the cell cycle is halted to gain necessary time for repairing DNA and genes required for repair and protection of other cellular components endangered by the genotoxic stress are activated. Some organisms do respond to the abiotic stresses by way of apoptosis i.e. eliminating the damaged cell [70]. On exposure to bleomycin (genotoxic), CDCP genes showed differential expression in root and shoot at all the time points except for the 6 hr in shoot, where none of the gene showed enhanced expression. This data indicates that the CDCPs might also play role in protecting the cells from the genotoxic stress. Under drought conditions, plants adapt themselves to maintain the cellular homeostasis. It has been observed that sensitive plants suffer rapid irreversible cell damage, essentially due to degradation of their membranes [71]. Membranes are main targets of degradative processes induced by drought and it has been shown that, under water stress, a decrease in membrane lipid content is correlated to the inhibition of lipid biosynthesis [72,73] and a stimulation of lipolytic and peroxidative activities [74-77]. When observed under drought conditions, all CDCP genes showed comparable expression at different time points in root, while in shoot approximately all the CDCP genes were found to be upregulated at 24 hr drought stress, which indicates that plant takes some time to adapt to the drought conditions, suggesting that CDCPs might also help plants to adapt to drought stress. Salt and osmotic stress result in transient increase in a cytoplasmic free calcium concentration, and disruption of this calcium gradient affects downstream gene expression [78]. Earlier, some attempts have been made to identify the osmotic stress related genes in *Arabidopsis *[79]. Under the condition of osmotic stress, expression of some of the CDCP genes was found to be altered with respect to the control in 30 min and 12 hr stress in roots, while in shoots these genes showed higher expression at 1 hr and 24 hr of osmotic stress. Under salt stress, CDCP genes were found to be up regulated at 1, 6 and 12 hr time points in roots, while in shoots these genes were expressed more in 1 hr and 24 hr of salt stress. The analysis of expression of these genes gives an idea that CBS domain containing proteins might play an important role in drought, salt and osmotic stress response/tolerance. Oxidative stress, arising from an imbalance in the generation and removal of reactive oxygen species (ROS) such as hydrogen peroxide (H_2_O_2_), is a challenge faced by all aerobic organisms [80,81]. Although ROS were originally considered to be detrimental to cells, it is now widely recognized that redox regulation involving ROS is a key factor modulating cellular activities [82]. Oxidative stress seems to induce the expression of CDCP genes at 3 hr of stress in roots while in shoot almost all the CDCP genes, which were differentially expressed showed upregulation under oxidative stress.

**Figure 10:**
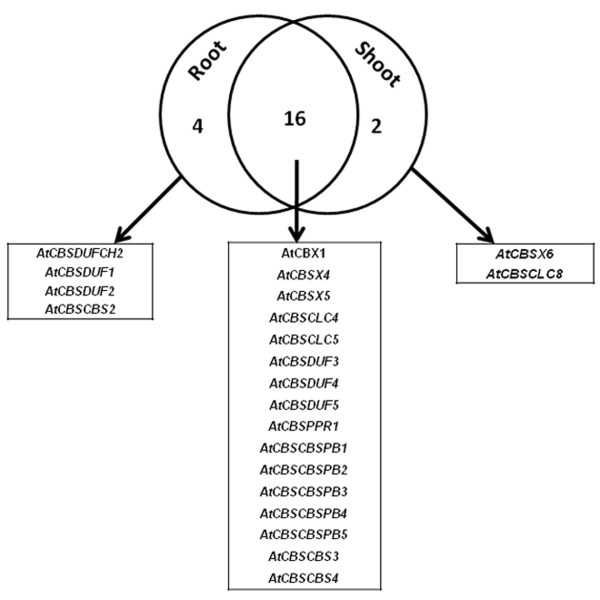
**Venn diagram depicting the complexities of tissue specific expression of CDCP genes in root and shoot tissues, under various stress conditions as revealed by microarray expression analysis in *Arabidopsis thaliana***.

This analysis revealed two important facts about the CDCP genes. On the one hand, we could identify some members (such as AtCBSX2, AtCBSX3, and AtCBSCBS1; three in total) whose expression was found to be unaltered under any stress conditions, in either roots or shoots. On the other hand, some genes could be identified (such as AtCBSX1 and others shown in Figure 10; sixteen in total) which were found to be differentially expressed with respect to the control under all the stress conditions in both, roots and shoots. Similarly, members such as AtCBSDUFCH2, AtCBSDUF1, AtCBSDUF2 and AtCBSCBS2 were altered under all stresses only in roots (four in total). In contrast, two genes (AtCBSX6 and AtCBSCLC8) could also be identified which were differentially expressed under all stresses only in shoots. It was also observed that more CDCP genes were differentially regulated under all stresses in roots tissues (20 in total) than in shoot tissues (18 in total).

## Conclusion

The cystathionine β-synthase (CBS) domain containing proteins (CDCPs) comprise of a large superfamily of evolutionarily conserved proteins which are present in all kingdoms of life. In plants, CDCPs were never reported and hence their occurrence and possible function is still a mystery. The present study has identified CDCPs in *Arabidopsis thaliana *and *Oryza sativa *at the whole genome level. We also propose their classification based on the presence of CBS domain and various other functional domain(s) present in them. The chromosomal position of genes encoding CDCPs gives an insight into their distribution in the whole genome of *Arabidopsis *and *Oryza*. In addition, MPSS analysis reveals the differential expression of these genes in various tissues and parts under stress conditions in these plant species. Moreover, microarray expression data gives an overview that the CDCPs might play an important role in stress response/tolerance in *Arabidopsis *under various stress conditions. Thus, it can be concluded that CDCP genes exhibit strict stress and developmentally regulated expression patterns in *Arabidopsis *and *Oryza*. Certainly, there is a need to functionally validate the role of these CDCPs which exhibit "induced expression patterns" under multiple stresses. Another major finding of this work is the observed expansion of CDCP gene family in *Oryza *as compared to *Arabidopsis*. This expansion has been noticed in *Oryza *primarily because of presence of relatively more cases of alternative splicing as well as gene duplications, possibly indicating towards the significant involvement of CDCPs in development and stress responses. This study will be helpful in commenting on the structural and functional aspects of these unexplored proteins with respect to their roles under various abiotic stresses. Tools of functional genomics based on transgenic approach can further help in testing the "candidature" of these proteins with defined features towards improving stress tolerance in crop plants. The leads provided here would also pave the way for elucidating the precise role of individual CDCP proteins in plants.

## Methods

### Search and analysis of CBS domain containing proteins

The CDCP protein sequences were retrieved from whole genome sequences of *Arabidopsis thaliana *(TIGR version 5 and TAIR 8) and *Oryza sativa *(TIGR version 6). The sequences obtained from TIGR were also cross checked with TAIR8 sequences for any new instances of CDCP proteins in *Arabidopsis*. The domain structure of the CBS was used to identify and classify the CDCP proteins using the TIGR *A. thaliana *genome sequence version 5.0 and *O. sativa *version 6.0. Profiles unique to the CBS domain were used to screen all predicted proteins using the HMMER software (version 2.3.2; . These unique profiles are for Pfam HMM of CBS domain (accession no. PF00571) [83]. We have used these profiles as default parameters in the hmmsearch program of the HMMER package [84]. All the significant hits having positive scores were selected for classification and were then examined individually for accessory domains that are usually present. This was accomplished by searching the sequences against the Pfam database (version 21.0) to map the known domains, such as Voltage_CLC, CorC_HlyC, SIS, PB1, DUF21 and IMPDH1 (see Fig. 2 for a schematic representation). This step, besides mapping accessory domains to the CBS provides the basis of classifying these proteins. The percentage values of the sequence similarity and identity within the groups were obtained using BLAST. The respective CDCP protein sequences were also cross checked from the TAIR8 database for presence of any additional alternative splice forms in *Arabidopsis*.

For convenience, we have assigned name to protein sequence according to the domains observed in the respective protein sequence, where At is for *Arabidopsis thaliana *and Os is for *Oryza sativa*. The chromosomal positions of the CDCP genes were obtained from TIGR version 6 for *O. sativa *and TIGR version 5, TAIR version 8 for *A. thaliana*, and plotted using Dia version 0.96.1, an open source gtk+ based diagram creation program.

### Sequence and phylogenetic analysis

Multiple alignment analyses were performed using MUSCLE (version 3.6) program [85]. The unrooted parsimonious tree was plotted using protpars and drawtree program of phylip package (version 3.66) [86] using default parameters. Similarity and identity values among the protein sequences were analyzed using standalone BLAST (version 2.2.15) [87]. The figures for final alignment were prepared using Jalview multiple sequence alignment editor [88].

### Expression analysis using MPSS database

Expression evidence from MPSS (Massively Parallel Signature Sequencing) tags was determined from the *Arabidopsis *MPSS project  mapped to *Arabidopsis *and *Oryza *gene models. The signature was considered to be significant if it uniquely identifies an individual gene and shows perfect match (100% identity over 100% length of the tag). The normalized abundance (tags per million, tpm) of these signatures for a given gene in a given library represents a quantitative estimate of expression of that gene.

The description of MPSS libraries in *A. thaliana *is: CAF – Callus, hardened tissue that forms to protect the exposed areas of cuttings; INF – Inflorescence, part of the plant that consists of flower bearing stalks; LEF – Leaves – 21 day, untreated, classic MPSS; ROF – Root – 21 day, untreated, classic MPSS; SIFSilique (Seedpod) – 24 to 48 hr post-fertilization, classic MPSS; AP1 – ap1-10 inflorescence (part of the plant that consists of flower bearing stalks) – mixed stage, immature buds; AP3 – ap3-6 inflorescence (part of the plant that consists of flower bearing stalks) – mixed stage, immature buds; AGM – agamous inflorescence (part of the plant that consists of flower bearing stalks) – mixed stage, immature buds; INS – Inflorescence – mixed stage, immature buds; ROS – Root – 21 day, untreated; SAP – sup/ap1 inflorescence – mixed stage, immature buds; S04 – Leaves, 4 hr after salicylic acid treatment; S52 – Leaves, 52 hr after salicylic acid treatment; LES – Leaves – 21 day, untreated; GSE – Germinating seedlings; CAS – Callus (hardened tissue that forms to protect the exposed areas of cuttings) – actively growing, signature MPSS; SIS – Silique (Seedpod) – 24 to 48 hr post-fertilization, signature MPSS.

The description of MPSS libraries in *O. sativa *is: NYR -14 days – Young Roots, NRA – 60 days – Mature Roots – Replicate A, NRB – 60 days – Mature Roots – Replicate B, NGD – 10 days – Germinating seedlings grown in dark, NST – 60 days – Stem, NYL – 14 days – Young leaves, NLA – 60 days – Mature Leaves – Replicate A, NLB – 60 days – Mature Leaves – Replicate B, NLC – 60 days – Mature Leaves – Replicate C, NLD – 60 days – Mature Leaves – Replicate D, NME – 60 days – Crown vegetative meristematic tissue, NPO – Mature Pollen, NOS – Ovary and mature stigma, NIP – 90 days – Immature panicle, NGS – 3 days – Germinating seed, NCA – 35 days – Callus, NSR – 14 days – Young roots stressed in 250 mM NaCl for 24 h, NSL – 14 days – Young leaves stressed in 250 mM NaCl for 24 h, NDR – 14 days – Young roots stressed in drought for 5 days, NDL – 14 days – Young leaves stressed in drought for 5 days, NCR– 14 days – Young roots stressed in 4 C cold for 24 h, NCL – 14 days – Young leaves stressed in 4 C cold for 24 h, 9RO – Roots, I9RR – Roots – Replicate, 9LA – Leaves, 9LB – Leaves – Replicate, 9LC – Leaves, 9LD – Leaves – Replicate, 9ME – Meristematic Tissue, FRO – F1 Hybrid 60 days Mature Root, FRR – F1 Hybrid 60 days Mature Root-Repl, FLA – F1 Hybrid 60 days Mature Leaf Replicate A, FLB – F1 Hybrid 60 days Mature Leaf Replicate B, FLC – F1 Hybrid 60 days Mature Leaf Replicate C, FLD – F1 Hybrid 60 days Mature Leaf Replicate D, FME – F1 Hybrid 60 days Meristematic tissue, PSC – rice developing seeds, 6 days old cypress high milling (99–1710), PSI – rice developing seeds,6 days old, Ilpumbyeo – High Taste, PSL – rice developing seeds, 6 days old, LaGrue-Low Milling, PSN – rice developing seed, 6 days old, Nipponbare-Grain quality control, PSY – rice developing seeds, 6 days old, Expression values obtained from MPSS database for respective CDCP genes were used for making the heatmap using gplots package of open source R software.

### Expression analysis using microarrays

The microarray data of the selected gene expression for various abiotic stress conditions such as cold, UV, wound, genotoxic stress, drought, osmotic, salt and oxidative stress were retrieved from the *Arabidopsis *Information Resource [89]. The datasets obtained were corresponding to root and shoot tissues at different time sets of stress namely 30 min, 1 hr, 3 hr, 6 hr, 12 hr and 24 hr. Fold increase in transcript abundance under stress conditions were calculated with respect to their controls. The transcript abundance with respect to the control was calculated using PERL scripts. The hierarchical clustering analysis and the heatmaps were made using gplots package of R software.

## Authors' contributions

SKS, SLS-P and AP made contributions to the conception of the study and in the preparation of the final draft of the manuscript. HRK, AKS and AP devised overall strategy, performed analysis, drafted and edited the manuscript. HRK developed relevant programs for sequence and microarray analysis. All authors read and approved the final manuscript.

## Supplementary Material

Additional file 1 **Multiple sequence alignment of amino acid sequence of single CBS domain in Arabidopsis thaliana and Oryza sativa.** Multiple sequence alignment of amino acid sequence of single CBS domain in Arabidopsis and Oryza. The alignments of the domain sequences were obtained using MUSCLE software and figures were prepared using multiple sequence alignment editor-Jalview. The consensus sequence is shown below these graphs. Click here for file

Additional file 2 **Multiple sequence alignment of amino acid sequence of two CBS domains in Arabidopsis thaliana and Oryza sativa.** Multiple sequence alignment of amino acid sequence of two CBS domains in Arabidopsis and Oryza. The alignments of the domain sequences were obtained using MUSCLE software and figures were prepared using multiple sequence alignment editor-Jalview. The consensus sequence is shown below these graphs. Click here for file

Additional file 3 **MPSS analysis of CBS domain containing proteins in *Oryza sativa***. Expression evidence from MPSS tags was determined from the *Oryza sativa *MPSS project . The normalized abundance (tags per million, tpm) of these signatures for a given gene in a given library represents a quantitative estimate of expression of that gene. The description of these libraries is given in methods. Click here for file

Additional file 4 **MPSS analysis of CBS domain containing proteins in A. thaliana. **Expression evidence from MPSS tags was determined from the Arabidopsis MPSS project http://mpss.udel.edu/at/. The normalized abundance (tags per million, tpm) of these signatures for a given gene in a given library represents a quantitative estimate of expression of that gene. The description of these libraries is given in methods. Click here for file
