# Supplementary figures and images for: Genome wide expression analysis of CBS domain containing proteins in Arabidopsis thaliana (L.) Heynh and Oryza sativa L. reveals their developmental and stress regulation
Source: BMC Genomics. 2009 Apr 28;10:200. doi: 10.1186/1471-2164-10-200 (PMC2694836; doi:10.1186/1471-2164-10-200)

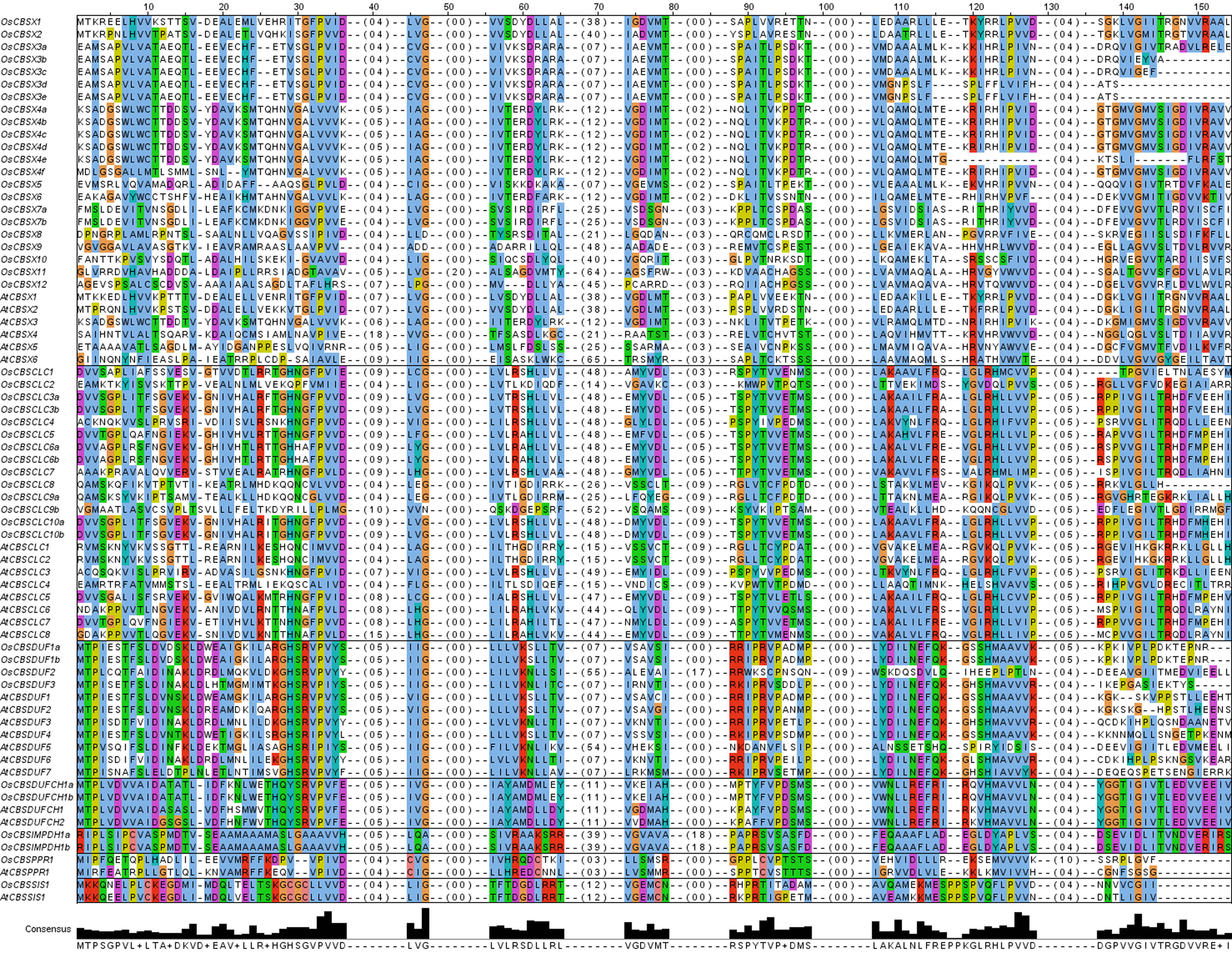

Supplement: Additional file 1 — Multiple sequence alignment of amino acid sequence of single CBS domain in Arabidopsis thaliana and Oryza sativa. Multiple sequence alignment of amino acid sequence of single CBS domain in Arabidopsis and Oryza. The alignments of the domain sequences were obtained using MUSCLE software and figures were prepared using multiple sequence alignment editor-Jalview. The consensus sequence is shown below these graphs. [file 1471-2164-10-200-S1.jpeg]

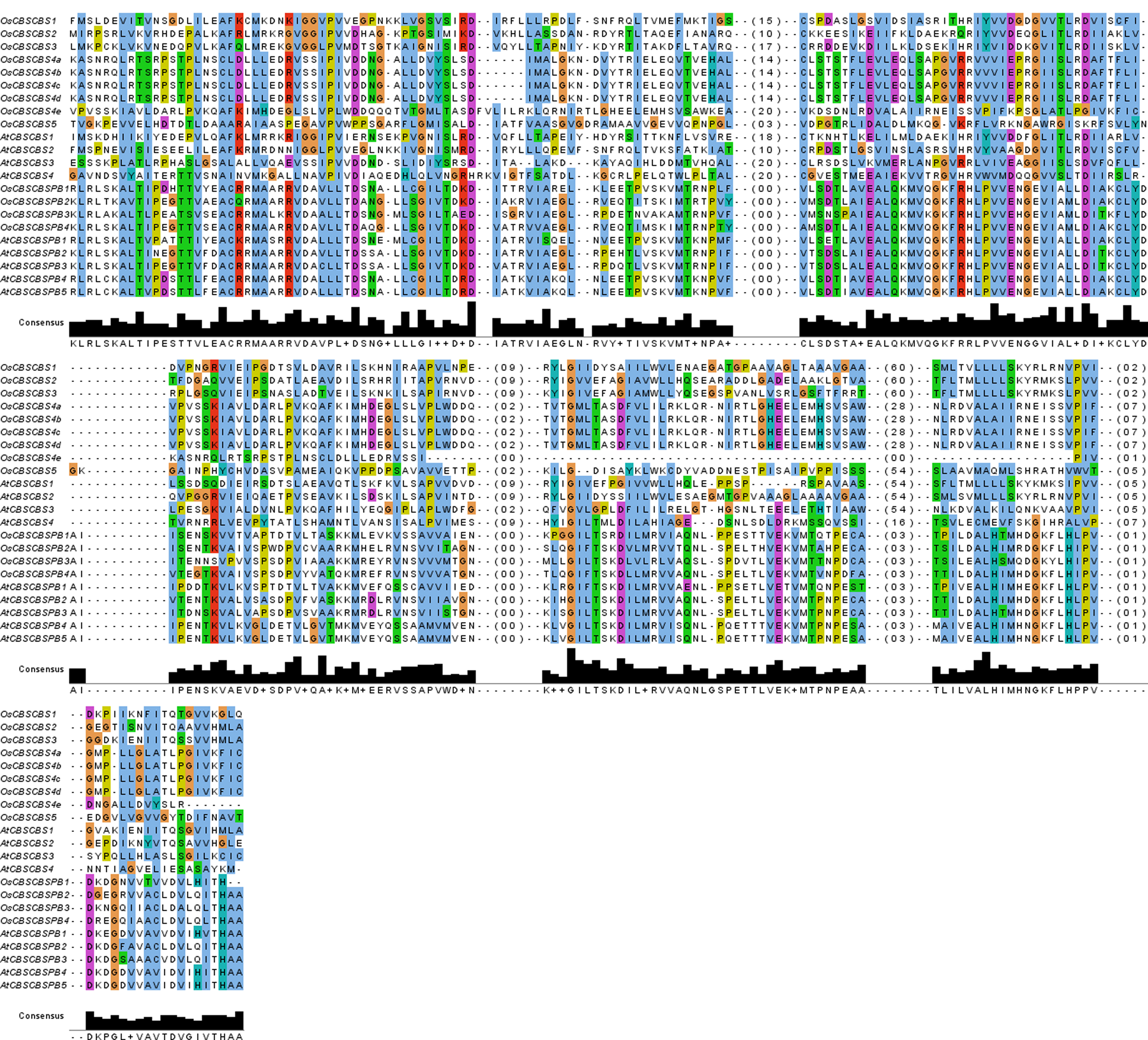

Supplement: Additional file 2 — Multiple sequence alignment of amino acid sequence of two CBS domains in Arabidopsis thaliana and Oryza sativa. Multiple sequence alignment of amino acid sequence of two CBS domains in Arabidopsis and Oryza. The alignments of the domain sequences were obtained using MUSCLE software and figures were prepared using multiple sequence alignment editor-Jalview. The consensus sequence is shown below these graphs. [file 1471-2164-10-200-S2.jpeg]
